# Effect of Polyphenol-Rich Dark Chocolate on Salivary Cortisol and Mood in Adults

**DOI:** 10.3390/antiox8060149

**Published:** 2019-05-29

**Authors:** Catherine Tsang, Lindsay Hodgson, Anna Bussu, Grace Farhat, Emad Al-Dujaili

**Affiliations:** 1 Faculty of Health and Social Care, Edge Hill University, St. Helen’s Road, Ormskirk, Lancashire L39 4QP, UK; 23272325@edgehill.ac.uk (L.H.); anna.bussu@edgehill.ac.uk (A.B.); 2 School of Health Sciences, Liverpool Hope University, Hope Park, Liverpool L16 9JD, UK; farhatg@hope.ac.uk; 3 Centre for Cardiovascular Science, University of Edinburgh, Queen’s Medical Research Institute, Edinburgh EH16 4TJ, UK; ealduja1@exseed.ed.ac.uk

**Keywords:** polyphenols, flavonoids, mood, stress, glucocorticoid, cortisol, Positive Affect and Negative Affect Schedule, dark chocolate

## Abstract

The aim of the present study was to investigate whether ingestion of polyphenol-rich dark chocolate improved salivary cortisol levels and subjective mood states in adults recruited from a health and social care setting. Twenty-six participants ingested 25 g/day of a high polyphenol dark chocolate (containing 500 mg of total flavonoids) or a similar amount of a control dark chocolate containing negligible flavonoids for four weeks. Twenty-four-hour salivary glucocorticoid levels (cortisol and cortisone) were measured by an enzyme-linked immunosorbent assay, and subjective mood was assessed using a validated Positive Affect and Negative Affect Schedule. Total daily cortisol, morning cortisol, and the cortisol/cortisone ratio were significantly reduced (*p* < 0.001) after ingestion of only the high polyphenol dark chocolate. There were no significant differences between groups for overall scores for positive affect and negative affect. No changes were observed after the control dark chocolate, or any other parameter measured. In conclusion, the findings from this small-scale study indicate lowering of salivary cortisol levels following polyphenol-rich dark chocolate in adults recruited from a health and social care setting. Such changes may be attributable to their ability to inhibit 11β-hydroxysteroid dehydrogenase type 1 activity and warrant further investigation.

## 1. Introduction

Chronic stress is an important risk factor for several psychophysical pathologies including cardiovascular disease (CVD), hypertension, insulin resistance, musculoskeletal illness, anxiety, and depression [1]. Work-related or occupational stress is increasingly prevalent in the UK population, and contributes to an increased health and economic cost, sickness absence, high staff turnover, and early retirement [2]. It is estimated that one in four people in the UK suffer from an anxiety-related illness each year, and over 49% of all sickness absences reported in 2016/17 was due to stress, depression, or anxiety [3]. Stress is associated with burn out syndrome (BOS), which occurs due to too much effort during a period of work with little recovery time, and can affect those across all types of work; however, high stress level occupations, such as healthcare professions, can lead to more BOS than lower stress level occupations, and these occupations have an adverse effect on mood, mental health, wellbeing, and overall quality of life [4,5].

Recent evidence of organisational stress in healthcare professions, such as medical, nursing, and support work, indicated a diverse range of work stressors beyond work volume alone and a lack of robust interventions to prevent and manage them [1]. Stress-related psychiatric syndromes, such as anxiety and depression, share common biological mechanisms that include the dis-regulation of the hypothalamic–pituitary–adrenal (HPA) axis [6,7,8,9,10]. In effect, the HPA axis is activated during the stress response increasing cortisol levels, and prolonged activation may contribute to the onset of mood deterioration and affective disorders including anxiety and depression [11]. Since prevention and management of risk factors linked to occupational stress are not yet adequately structured, and with no measure of long-term effectiveness on healthcare professions, it is essential to explore alternative strategies which are modifiable and easily accessible.

Polyphenols are a diverse and heterogeneous group of secondary plant metabolites, including phenolic acids, flavonoids, stilbenes, and lignans found in many fruits, vegetables, and beverages in the human diet, where dietary intake levels have been estimated to be in the region of 1 g/day [12]. Flavonoids represent one of the largest groups of natural phenols thought to exert putative health benefits through cell-mediated signaling pathways, antioxidant, anti-inflammatory, neurological, and cardiovascular effects [13,14,15,16]. There is limited evidence of the impact of flavonoids on stress. Nonetheless, studies in chronically stressed rats indicate their ability to improve hippocampal dysfunction [17] and lower corticosterone and adrenocorticotropic hormone (ACTH) levels [18]. Other studies have shown the ability of flavonoids to moderate anxiety by binding to benzodiazepine sites on gamma-amino butyric acid (GABA) (A)-receptors and exert antidepressant effects by inhibiting monoamine oxidase (MOA) [19]. Human studies have reported anxiolytic properties of flavonoids in black and green tea [20]. Cocoa-derived products, including dark chocolate (DC), have demonstrated some benefit when used as an adjunct to antidepressant treatment [21], while anxiety and depressive symptoms were reduced in those with chronic fatigue [22]. Other human studies have indicated a possible role in their ability to counter mood deterioration and improve positive mood state following ingestion of blueberries [23] and cocoa, especially at dosages of ≥ 520 mg total flavonoids [24,25].

Flavonoids may influence the HPA axis by reducing cortisol levels, which could influence physiological stress; however, it is uncertain whether these effects translate to psychological stress and wellbeing, especially in populations prone to high levels of occupational stress, such as those in healthcare settings.

Therefore, the aim of the present study was to conduct an exploratory investigation on the effect of polyphenol-rich dark chocolate (DC) on salivary glucocorticoid (GC), cortisol and cortisone, and on the self-reported subjective mood in health and social care professionals.

## 2. Materials and Methods

### 2.1. Participants

All study participants were recruited from the Faculty of Health and Social Care at Edge Hill University, UK, in response to an internal email and poster recruitment moderator.

Participants of thirty males and females aged between 23 and 55 years volunteered to take part in the study. The eligibility criteria included (a) healthy males and females; (b) aged ≥18 years; (c) nonsmokers; (d) not taking dietary and antioxidant supplements; (e) no history of, and not taking regular medication for, heart disease, hypertension, liver or kidney disease, high cholesterol, autoimmune disease, cancer, psychiatric disorders, or diabetes; (f) no history of, and not taking regular medication for, any pulmonary, thyroid, neuromuscular or neurological condition; (g) not pregnant or breastfeeding; and (h) no food allergies or food intolerances.

The research ethics committee at Edge Hill University, UK, approved the study (code: URESC17-LH01), which conformed to the guidelines set by the Declaration of Helsinki. All participants were provided with information on the purpose of the research and experimental procedures, and written informed consent was obtained.

### 2.2. Study Design

The study followed a single-blind parallel design over 4 weeks and participants were randomly allocated to receive a daily intake of a 25 g serving of high polyphenol dark chocolate (HPDC), which contained 500 mg flavonoids, or a similar serving of a low polyphenol dark chocolate (LPDC) containing negligible flavonoids. A health questionnaire was used to screen for any health condition(s) and to assess eligibility. All participants were asked to refrain from consuming foods and beverages known to contain high amounts of polyphenols, such as green tea, black tea, coffee, red wine, DC, and berries, which could interfere with the study DC for the duration of the study period.

Participants recorded food intake using a three-day estimated food diary completed over two weekdays and one day over the weekend, at the beginning and at the end of the study period to monitor compliance. A sample size of twenty-eight participants with 80% power and a 0.05 two-sided significance level was needed to detect an effect size of 0.25. Assuming 5% attrition, thirty participants were recruited. Four participants who met the inclusion criteria failed to complete the study mainly due to a lack of time and/or inability to commit to the study protocol, and twenty-six participants completed the study.

### 2.3. Experimental Procedures

Participants attended the university on three separate occasions; at the start, in the middle and at the end of the study period, separated by two weekly intervals. Each appointment lasted 30 min (between 9:00 a.m. and 1:00 p.m.). Height (m) and weight (kg) were measured for body mass index (BMI), and an automated A&D Medical UA-767 BP monitor (A&D Medical, San Jose, CA, USA) was used to monitor arterial blood pressure (BP), in accordance with previous methods [26]. Subjective mood was assessed using a validated Positive and Negative Affect Schedule (PANAS) [27]. The PANAS questionnaire contained 20 words including active, alert, attentive, determined, enthusiastic, excited, inspired, interested, proud, and strong relating to positive affect (PA), while afraid, scared, nervous, jittery, irritable, hostile, guilty, ashamed, upset, and distressed were related to negative affect (NA). These were marked on a five-point Likert scale with one being ‘very slightly or not at all’ and five being ‘extremely’. Participants were asked to score each emotion based on their experience of these over the previous week, and the sum of each was used to provide an overall PA and overall NA score between 10 and 50. Participants collected their own saliva samples into labeled plastic tubes following written instructions and asked to refrain from strenuous exercise and alcohol consumption for 24 h prior to providing a sample. Saliva was collected over a 24 h period (morning, midday, and evening) at baseline, 2 and 4 weeks post-ingestion of the DC. Samples were stored between ca. 4–5 °C until their appointment, after which samples were stored at −80 °C until processed and analysed by an enzyme-linked immunosorbent assay (ELISA) in accordance with previous methods [28].

Barry Callebaut (Zurich, Switzerland) provided the study chocolate which were stored in the dark at 5 °C throughout the study period. The nutrient composition of the DC was provided by the supplier and each 25 g serving of HPDC contained 135 kcal, 9.7 g carbohydrate, 2 g protein, 9.2 g fat, 2 g fibre, and 8.1 g sugars. Each 25 g serving of LPDC contained 137 kcal, 11.3 g carbohydrate, 1.3 g protein, 9.2 g fat, 2 g fibre, and 10.7 g sugars. The HPDC contained 500 mg of total flavonoids per 25 g serving or 2% total flavonoids and 65.7% of cocoa solids, while the LPDC contained negligible flavonoids and 56% of cocoa solids. The dosage of 500 mg of total flavonoids was selected based on the suggested optimal dosage for cocoa flavonoids, based on existing literature from human studies, assessing their effect on mood [24,25]. In addition, we also followed guidance from the supplier of the chocolate regarding the possibility of alterations to taste, texture, and acceptability (i.e., enhanced bitterness) with doses more than 500 mg. The control DC was matched for taste, texture, and colour and contained a similar nutrient composition to the HPDC, albeit negligible flavonoids.

Participants were provided with instruction to ingest their DC dose throughout the day and to maintain their usual dietary intake. Food diaries were analysed for energy and macronutrient intake using Nutrition Analysis Software V5.042 (Nutritics Ltd., Dublin, Ireland). Compliance with the study protocol was assessed by direct interviewing during each appointment at the university and assessment of the food diaries.

### 2.4. Data Processing, Analyses, and Statistics

The mean values and standard deviations were calculated for each variable, and SPSS (Statistical Package for the Social Sciences, version 21, Chicago, IL, USA) was used to analyse the data. A mixed model analysis of variance (ANOVA) was performed to evaluate the differences between times at baseline, 2 weeks, and 4 weeks, with treatment of HPDC and LPDC, and comparisons were used with Bonferroni’s test to determine significance, which was set at *p* ≤ 0.05.

## 3. Results

### 3.1. Anthropometric Indices and Blood Pressure

Table 1 shows the effect of high polyphenol dark chocolate (HPDC) and low polyphenol dark chocolate (LPDC) on anthropometric indices, body mass index (BMI) (kg/m^2^) and body mass (kg), and on blood pressure (BP), systolic (SBP) and diastolic (DBP), measures in 26 male and female participants (age range: 23–55 years; mean age: 38.8 ± 11.1 years; mean BMI: 26.8 ± 5.9 kg/m^2^). There were no significant differences between mean age (years) and body mass (kg), and no changes were observed in dietary intake for total fat, carbohydrate, protein or total energy intake (data not shown). As for BMI, the assumption of sphericity was violated and a Greenhouse-Geisser correction was applied (epsilon (ε) = 0.51). There were no significant interactions between treatment and time on BMI (F (1.01, 48) = 0.32, *p* = 0.73), and there was no significant effect of time on BMI levels (F (1.01, 48) = 0.47, *p* = 0.63). There were also no significant interactions between treatment and time on SBP (F (2, 48) = 0.53, *p* = 0.59) and DBP (F (2, 48) = 1.76, *p* = 0.18).

### 3.2. Glucocorticoid Levels

**Figure 1:**
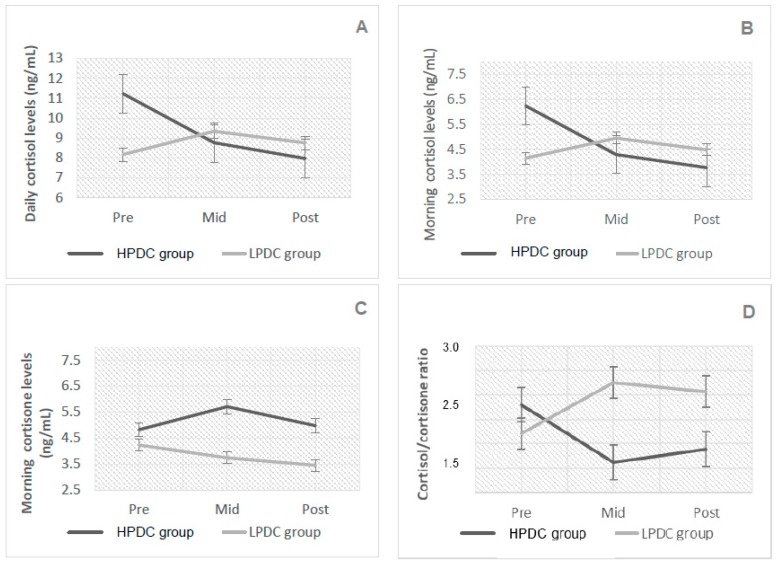
Salivary glucocorticoid measures at baseline, 2 weeks, and 4 weeks following HPDC and LPDC (mean values ± standard deviation); (**A**) Daily cortisol (ng/mL), *p* < 0.001; (**B**) morning cortisol (ng/mL), *p* < 0.001; (**C**) morning cortisone (ng/mL), n.s.; (**D**) cortisol/cortisone ratio, *p* < 0.001.

Figure 1 presents the cortisol and cortisone levels, and the cortisol/cortisone ratio for the HPDC and LPDC groups at baseline, 2 weeks, and 4 weeks, respectively. There was a significant effect of treatment and time on total daily cortisol levels (F (2, 48) = 11.24, *p* < 0.001) (Figure 1A), following HPDC only. Cortisol levels significantly decreased from baseline (11.23 ± 3.33 ng/mL) to week 4 (7.97 ± 3.42 ng/mL, *p* < 0.0001) in this group, while no significant difference between baseline and week 2 were noted (*p* > 0.05). There was also a significant effect of treatment and time on morning cortisol levels (F (2, 48) = 12.98, *p* < 0.001) (Figure 1B), which significantly decreased at week 2 (from 6.24 ± 1.54 ng/mL to 4.3 ± 1.62 mg/mL, *p* < 0.0001), while no significant difference was noted between week 2 and week 4 (*p* > 0.05). The cortisol/cortisone ratio also significantly decreased following HPDC only (F (2, 48) = 11.00, *p* < 0.001) (Figure 1D) at week 2 and week 4 (*p* < 0.0001 and *p* = 0.015, respectively). There was no significant effect of treatment and time on cortisone levels (F (1.62, 48) = 2.81, *p* = 0.08) (Figure 1C).

### 3.3. Subjective Mood (PANAS)

**Figure 2:**
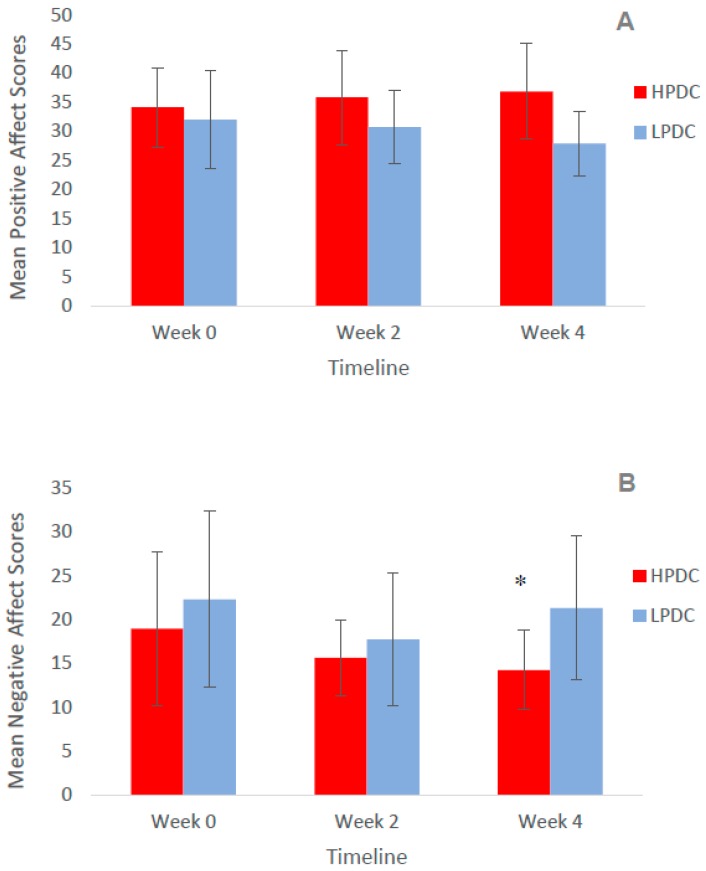
Mean Positive and Negative Affect Schedule (PANAS) scores for positive affect (PA) and negative affect (NA) at baseline, 2 weeks, and 4 weeks following HPDC and LPDC (mean values ± standard deviation); (**A**) mean PA score, n.s.; (**B**) mean NA score, n.s. * Significant effect of treatment and time on overall NA (*p* = 0.02) within the HPDC group after 4 weeks.

Figure 2 presents the overall scores for PANAS for the HPDC and LPDC groups at baseline, 2 weeks, and 4 weeks. There was no significant effect of treatment and time on the overall scores for PA (F (2, 48) = 2.12, *p* = 0.13) and overall scores for NA (F (2, 48) = 2.08, *p* = 0.14) (Figure 2A,B). Within groups, there was a significant effect of treatment and time on overall NA (F (2, 48) = 5.02, *p* = 0.01) following HPDC, with improvement in overall scores after 4 weeks, compared to baseline (mean difference = 1.47 (0.87, 3.82 CI), *p* = 0.02). There were no significant changes in NA in the LPDC group (mean difference = 1.0 (5.5, 7.5 CI), *p* = 1.00). No other significant differences were observed.

## 4. Discussion

The purpose of the present study was to investigate the effect of polyphenol-rich dark chocolate (containing 500 mg of total flavonoids) on salivary cortisol levels and subjective mood states, specifically PA and NA in adults recruited from a health and social care setting. Our findings indicate a lowering of salivary GC, specifically total daily cortisol, morning (or waking) cortisol and the cortisol/cortisone ratio following HPDC ingestion for 4 weeks. Cortisol is a GC hormone secreted by the adrenal cortex in response to several stimuli such as stress and inflammation [29,30]. Raised GC levels, which occur under conditions such as chronic stress, are associated with a range of psychophysical pathologies, including the metabolic syndrome and CVD, via their effect on the liver to enhance glucose, fat accumulation and glucose-dependent insulin insensitivity [31]. Chronic stress is often experienced in many high stress level occupations such as healthcare professions, which could lead to adverse effects not only on physical pathologies, but also on psychological conditions affecting mood, mental health and wellbeing, and overall quality of life [4,5]. Several stress-related psychiatric syndromes, including anxiety and depression, are in part due to the dis-regulation of the hypothalamic–pituitary–adrenal (HPA) axis [6,7,8,9,10]. Reductions in stress hormone levels, such as cortisol, have been associated with improving the regulation of the HPA axis [32], and flavonoids including those commonly found in the human diet, including cocoa-derived products such as DC, could be important in their ability to lower the levels of the active hormone cortisol [33]. Evidence demonstrates the ability of flavonoids to inhibit 11β-hydroxysteroid dehydrogenase (11β-HSD) type 1, an enzyme involved in reducing cortisone to the active form cortisol [34]. Zhu et al. [35] demonstrated an increasing potency in their level of inhibition of this enzyme for the flavonoids apigenin, quercetin, and genistein, respectively and confirmed their mode of action as noncompetitive inhibitors of human 11β-HSD type 1 reductase. In the present study, the inhibition of 11β-HSD type 1 was indicated by the reduction in the ratio of free cortisol to free cortisone. The ratio of cortisol to cortisone is well accepted by many researchers as indicative of 11β-HSD type 1 activity [29,36].

According to Watson et al. [27], a low score for PA is associated with conditions related to depression while a high score for NA is associated with those related to anxiety. There were no significant effects observed for overall scores for PA and NA in the present study. To our knowledge, the association between mood and stress is a proposed mechanism, however we did not find any correlation to corticosterone changes in the present study. There is limited evidence on the effect of flavonoids on mood states such as PA and NA and further work is needed. There were several limitations to the present study. This was a small-scale study and the sample size was small due to the exploratory nature of the study. We observed a significant difference between cortisol levels at baseline in the HPDC group. Salivary cortisol represents the free fraction of the hormone, which is the active form, and therefore, small changes in salivary cortisol may have marked biological effects. It is uncertain as to why the basal cortisol in the LPDC group is lower than the HPDC group. Cortisol is a stress hormone and the values are highly variable depending on several factors, which influence their levels. These differences are to be expected. Therefore, we determined the ratio of cortisol to cortisone in order to overcome these differences. The ratio was found to be significant (i.e. lower after the HPDC). This aspect is important as it indicates less activity of cortisol. In this regard, further studies are necessary to elucidate this. Most of our study participants were female (*n* 18), which potentially may have influenced our findings. Nonetheless, a recent study by Khalid et al. [23] investigated the effect of blueberry polyphenols on subjective mood and observed significant improvements in overall scores for PA. Their research also involved a small sample size (*n* 21), in predominantly young female adults (*n* 19). Our findings may not be generalisable to a male population; however, there is no evidence to suggest a gender-specific mechanism underlying the influence of flavonoids [23].

## 5. Conclusions

In conclusion, the findings from this small-scale study indicate lowering of salivary cortisol levels following polyphenol-rich dark chocolate in adults recruited from a health and social care setting. Such changes may be attributable to their ability to inhibit 11β-HSD type 1 activity, however future studies are warranted to interpret their precise role.

## Figures and Tables

**Table 1:** Anthropometric indices and blood pressure measures at baseline, 2 weeks, and 4 weeks following HPDC and LPDC (mean values ± standard deviation).

Variable	HPDC Group			LPDC Group		
	Pre	Mid	Post	Pre	Mid	Post
Body mass (kg)	73.4 ± 17.9	69.2 ± 24.3	73.3 ± 17.5	75.4 ± 23.7	75.2 ± 23.6	76 ± 24.5
BMI (kg/m^2^)	26.8 ± 5.8	25.1 ± 8.3	26.8 ± 5.6	27.2 ± 6.7	27.0 ± 6.6	27.1 ± 6.8
SBP (mmHg)	106.7 ± 9.2	106.5 ± 9.9	107.6 ± 13.1	97.8 ± 8.8	102.4± 9.7	99.6 ± 7.7
DBP (mmHg)	69.4 ± 7.5	72.3 ± 6.9	72.2 ± 7.1	65.6 ± 10.7	72.8 ± 8.6	68.3 ± 6.3

BMI: Body Mass Index, n.s.; DBP: Diastolic Blood Pressure, n.s.; SBP: Systolic Blood Pressure, n.s.; HPDC: High Polyphenol Dark Chocolate; LPDC: Low Polyphenol Dark Chocolate. Data were analysed using SPSS (21, Chicago, IL, USA).

## Abbreviations

ACTH	Adrenocorticotropic hormone
BMI	Body mass index
BP	Blood pressure
BOS	Burn out syndrome
CVD	Cardiovascular disease
DBP	Diastolic blood pressure
DC	Dark chocolate
ELISA	Enzyme-linked immunosorbent assay
GABA	Gamma-amino butyric acid
GC	Glucocorticoid
11β-HSD	11β-hydroxysteroid dehydrogenase
HPA	Hypothalamic–pituitary–adrenal axis
HPDC	High polyphenol dark chocolate
LPDC	Low polyphenol dark chocolate
MOA	Monoamine oxidase
NA	Negative affect
PANAS	Positive affect and negative affect schedule
PA	Positive affect
SBP	Systolic blood pressure
